# Pantoea Species Bacteremia in a Child With Sickle Cell Disease: A Case Report

**DOI:** 10.7759/cureus.55122

**Published:** 2024-02-28

**Authors:** Ali A Aljameely, Fawaz M AlZubaidi, Fayza I AlSiny, Fatima S Alzahrani, Kholoud A Hothan

**Affiliations:** 1 Department of Pediatrics, King Abdulaziz University, Jeddah, SAU

**Keywords:** immunocompromised hosts, gram-negative bacteremia, pica, pantoea species, sickle cell disease

## Abstract

The *Pantoea *genus of bacteria is a group of Gram-negative rod-shaped bacteria in the Enterobacteriaceae family. It is an uncommon cause of infection in humans except in specific settings, including hospital-acquired infections and in immunocompromised patients.

In this report, we describe the case of a 12-year-old girl with sickle cell disease who presented with a picture of sepsis and was found to have *Pantoea *species in her blood culture which was treated with antibiotics with a good response.

From our literature review, risk factors were identified in the reported cases, for which further exploration is highly recommended.

## Introduction

The *Pantoea *genus is a diverse group of Gram-negative rod-shaped bacteria belonging to the Enterobacteriaceae family. *Pantoea *species are usually isolated from water sources (e.g., rivers and thermal springs) and earthly environments (e.g., plants and soils), as well as from humans, animals, and insects. The site of isolation depends on each specific strain and its commonly known sources. *Pantoea *currently has 20 recognized strains that are phenotypically similar, with the strain *Pantoea agglomerans* being the most reported cause of infections in humans [1].

*Pantoea *species are an uncommon cause of infection in humans, except in some specific settings, as they have been found in hospital-acquired infections resulting in sepsis in both pediatric [2,3] and adult patients [4,5]. They have also been shown to cause septic arthritis or synovitis in plant-thorn injury cases, which usually occur during agricultural work, children playing, or in immunocompromised patients [6-11].

Sickle cell disease (SCD) is a group of genetic-hematological conditions characterized by a gene mutation in hemoglobin, resulting in sickle hemoglobin (Hb S) with distinctive sickled-shaped red blood cells. Due to their extreme viscosity, these asymmetrical blood cells might be clogged within the blood vessels at various body sites, impeding or slowing the blood flow to different organs [12].

Individuals diagnosed with SCD are considered immunocompromised due to functional asplenia, caused by vaso-occlusion of splenic vessels that usually begins in infancy [13,14], placing them at a higher risk of life-threatening infections.

Through a review of the literature, we found that the incidence of *Pantoea *infection in patients with SCD has only been reported three times [15-17]. Therefore, this is the fourth report of a patient with SCD who was found positive for *Pantoea *spp. in the blood culture.

## Case presentation

We present the case of a 12-year-old girl with sickle cell anemia (confirmed based on hemoglobin electrophoresis), complicated by Moyamoya syndrome (based on brain magnetic resonance imaging (MRI) results), with a previous history of three episodes of cerebrovascular accidents and multiple episodes of vaso-occlusive crisis. She was on hydroxyurea, folic acid, deferasirox, and regular exchange transfusion. The patient presented to the emergency department (ED) at King Abdulaziz University Hospital in Jeddah, Saudi Arabia, with a history of subjective fever for the last two days. The fever was associated with headache, non-projectile vomiting of food, along with decreased oral intake, and tea-colored urine. There was no history of abnormal movements or loss of consciousness, no other urinary symptoms, and no change in bowel habits.

On presentation, the patient was febrile with a temperature of 38.6°C, tachycardic with a heart rate of 125 beats per minute, and normotensive with a blood pressure of 118/70 mmHg. She looked ill, drowsy, and jaundiced, but was not in respiratory distress. Neurological examination of the patient was at baseline as previously recorded, with hypertonia and hyperreflexia of the right upper and lower limbs, 4/5 power in both upper and lower limbs, and normal left upper and lower limbs. Kernig’s and Brudzinski’s signs were negative. The rest of her examination was unremarkable.

The results of the laboratory workup done at the presentation are summarized in Table 1. Tests included complete blood count with differential which indicated hemolysis in the form of low hemoglobin (her baseline hemoglobin was 8.5-9.7 g/dL), high reticulocyte count, high lactate dehydrogenase, high bilirubin levels, along with leukocytosis, mainly neutrophilia. C-reactive protein was not available. The patient had high urea, which was attributed to dehydration, with a normal creatinine level. Liver function tests were within the normal range except for a high bilirubin level. Electrolytes were within the normal range.

**Table 1:** Laboratory test results of the patient at presentation.

Test name	Result	Reference range
Complete blood count		
Hemoglobin	7.3 g/dL	11.9–14.8 g/dL
Reticulocyte	19%	0.90–1.49%
Leukocyte	38.7 K/μL	3.8–10.4 K/μL
Neutrophil	29.6 K/μL, 76%	1.5–6.5 K/μL
Lactate dehydrogenase	565 U/L	120–293 U/L
Bilirubin		
Total	119 μmol/L	5.1–17 μmol/L
Direct	15 μmol/L	1.7–5.1 μmol/L
Urea	7.5 mmol/L	2.5–6.5 mmol/L

Blood and urine cultures were collected. Computed tomography of the brain was done and no new results were seen. A lumbar puncture was done and the sample was sent for cerebrospinal fluid (CSF) analysis and culture.

The patient was admitted as a case of SCD with hemolytic crisis and fever to rule out sepsis. She was started empirically on intravenous (IV) ceftriaxone and vancomycin, with IV fluid and IV paracetamol PRN for the fever. CSF test results were unremarkable (cell count: white blood cell, 1 cell/mm^3^; red blood cell, <1 cell/mm^3^; protein, 0.19 g/L; glucose, 5.5 mmol/L; Gram stain was negative). Vancomycin was discontinued after the CSF test results. The patient was kept on ceftriaxone and received a packed red blood cell transfusion.

On the second day of admission, the preliminary report of the blood culture came back positive for a Gram-negative bacillus. Therefore, the antibiotic was changed from IV ceftriaxone to IV meropenem, with the guidance of the infectious diseases team.

On the third day of admission, urine and CSF cultures came back negative after two days of incubation, and new blood culture samples were collected and sent for analysis.

On the fourth day of admission, the final results of the first blood culture came back, identifying the Gram-negative bacillus as *Pantoea *spp. (BacT/ALERT Virtuo; bioMérieux). The sensitivity of the organism and minimum inhibitory concentration of different antibiotics are shown in Table 2 (VITEK 2; bioMérieux).

**Table 2:** Antibiotic sensitivity and MIC. MIC: minimum inhibitory concentration

Antibiotic	MIC	Sensitivity
Meropenem	≤1	Sensitive
Ciprofloxacin	≤0.25	Sensitive
Cefepime	≤2	Sensitive
Gentamicin	≤4	Sensitive
Imipenem	≤1	Sensitive
Trimethoprim/Sulfa	≤40	Sensitive

The patient’s condition improved over the first three days of antibiotic therapy, following which she was afebrile. She completed a course of meropenem for 14 days. Counting from the first negative blood culture, the treatment was for a total of 17 days.

## Discussion

The reported cases in the literature of *Pantoea *species infection were observed in individuals with specific risk factors, including immunocompromised individuals [4,5,18], nosocomial infections [2,3,5,19,20], and plant-thorn injuries [6-11].

Upon further assessment of the risk factors in our patient, we observed that, in addition to being immunocompromised, an indication of chronic anemia put her at risk of pica; regarding this, the patient admitted to only having pagophagia, but no history of soil or plant ingestion. On further exploration of the patient’s medical history, she reported a history of plant-thorn injury while playing outside that occurred five months before her current presentation.

Interestingly, two days before her presentation, there had been floods in the area she was living in. The patient admitted to playing in the rain and drinking rainwater, after which she started to develop a fever at night.

In the previously reported cases of patients with SCD infected with *Pantoea *[14-16], the possibility of gut translocation of the bacteria was raised, as one of the three reported cases was of a pregnant lady with SCD having a history of long-standing geophagia (i.e., potting soil consumption). The second case was of a child with a history of soil ingestion who was found to have *Pantoea* bacteremia.

As the environmental exposure in our case extended beyond the plant-thorn injury to include playing in flooded areas and drinking rainwater, this aspect of the patient’s history underscores the potential gastrointestinal route of translocation for *Pantoea *infections.

## Conclusions

To our knowledge, this is the fourth reported case of *Pantoea *infection in patients with sickle cell anemia. This case report contributes to the growing body of evidence linking *Pantoea *infections to immunocompromised states and environmental exposure.

As we continue to uncover the intricacies of *Pantoea *infections, future research should focus on elucidating the specific mechanisms of transmission, identifying additional risk factors, and refining treatment strategies for this uncommon but clinically significant bacterial genus in susceptible patient populations.
